# K_Ca_3.1 Channels Confer Radioresistance to Breast Cancer Cells

**DOI:** 10.3390/cancers11091285

**Published:** 2019-09-01

**Authors:** Corinna J. Mohr, Dominic Gross, Efe C. Sezgin, Friederike A. Steudel, Peter Ruth, Stephan M. Huber, Robert Lukowski

**Affiliations:** 1 Department of Pharmacology, Toxicology and Clinical Pharmacy, Institute of Pharmacy, University of Tuebingen, 72076 Tuebingen, Germany; 2 Dr Margarete Fischer-Bosch-Institute of Clinical Pharmacology, 70376 Stuttgart and University of Tuebingen, 72076 Tuebingen, Germany; 3 Department of Radiation Oncology, University of Tuebingen, 72076 Tuebingen, Germany

**Keywords:** breast cancer, ionizing radiation, intermediate conductance calcium-activated potassium channel, K_Ca_3.1, SK4, IK, KCNN4, mouse mammary tumor virus polyoma middle T antigen, MMTV-PyMT, TRAM-34

## Abstract

K_Ca_3.1 K^+^ channels reportedly contribute to the proliferation of breast tumor cells and may serve pro-tumor functions in the microenvironment. The putative interaction of K_Ca_3.1 with major anti-cancer treatment strategies, which are based on cytotoxic drugs or radiotherapy, remains largely unexplored. We employed K_Ca_3.1-proficient and -deficient breast cancer cells derived from breast cancer-prone MMTV-PyMT mice, pharmacological K_Ca_3.1 inhibition, and a syngeneic orthotopic mouse model to study the relevance of functional K_Ca_3.1 for therapy response. The K_Ca_3.1 status of MMTV-PyMT cells did not determine tumor cell proliferation after treatment with different concentrations of docetaxel, doxorubicin, 5-fluorouracil, or cyclophosphamide. K_Ca_3.1 activation by ionizing radiation (IR) in breast tumor cells in vitro, however, enhanced radioresistance, probably via an involvement of the channel in IR-stimulated Ca^2+^ signals and DNA repair pathways. Consistently, K_Ca_3.1 knockout increased survival time of wildtype mice upon syngeneic orthotopic transplantation of MMTV-PyMT tumors followed by fractionated radiotherapy. Combined, our results imply that K_Ca_3.1 confers resistance to radio- but not to chemotherapy in the MMTV-PyMT breast cancer model. Since K_Ca_3.1 is druggable, K_Ca_3.1 targeting concomitant to radiotherapy seems to be a promising strategy to radiosensitize breast tumors.

## 1. Introduction

Due to their reported contribution to various processes in cancerogenesis and malignant progression, K_Ca_3.1 channels are being increasingly explored in several cancer entities [1,2] such as leukemia [3,4], glioblastoma [5,6] as well as in gynecological cancers [7,8]. In the latter, K_Ca_3.1 has been described in particular for its role in breast cancer where its expression depends on cell cycle and where K_Ca_3.1 contributes to Ca^2+^ signaling and G_1_/S transition. Recently, genetic ablation or pharmacological inhibition of the K_Ca_3.1 has been shown to interfere with proliferation of breast tumor cells in vitro and to prolong survival of an orthotopic breast cancer mouse model [9,10].

Breast cancer is the most common cancer entity in women, and, despite general advances and targeted therapies, it is still associated with high mortality [11,12,13]. Hence, the identification of novel markers prognostic for disease progression or predictive for therapy response as well as novel potential therapeutic targets are urgently required. Standard treatment regimens of breast cancer include chemo- and radiotherapy. Notably, the abundance of K_Ca_3.1 in the breast tumor has been associated with enhanced treatment efficacy and sensitivity to cisplatin [14], presumably via the contribution of K_Ca_3.1 to the cellular uptake of cisplatin [15]. In contrast, pharmacological blockade of K_Ca_3.1 with TRAM-34 sensitizes glioblastoma cells to the DNA-alkylating agent temozolomide (TMZ), which is used for standard therapy of malignant glioma [16]. In that study, TRAM-34 reduced glioma cell migration and invasion, and increased apoptosis. A further report on the function of K_Ca_3.1 in glioma therapy resistance suggests a direct inhibition of the channel by TMZ pointing to the possibility that TMZ exerts part of its anti-neoplastic action via inhibition of K_Ca_3.1 [17]. Besides chemotherapy, K_Ca_3.1 has been implied to contribute to radioresistance of lung adenocarcinoma and glioblastoma [18,19]. Accordingly, pharmacological inhibition of K_Ca_3.1 opposed the pro-migratory and pro-invasive behavior of patient-derived glioblastoma cells, which is frequently seen in radiation therapy-resistant tumors [20].

We used cells isolated from a genetic breast cancer mouse model derived from mouse mammary tumor virus (MMTV)-mediated overexpression of the polyoma middle T antigen (PyMT) as oncogene. K_Ca_3.1 expression and contribution to breast cancer in the MMTV-PyMT model is evident from our previous studies [10]. To define the role of K_Ca_3.1 in therapy resistance of breast cancer, the response of MMTV-PyMT breast cancer cells to chemo- and radiotherapy was analyzed in vitro and in an orthotopic and syngeneic mouse model in dependence on wildtype (WT) versus knockout (KO) genotype or pharmacological blockade of K_Ca_3.1.

## 2. Results

### 2.1. Anti-Proliferative Effects of Cytotoxic Drugs Occur Independently from K_Ca_3.1 

In vitro chemotherapy was carried out with different concentrations of cytotoxic drugs commonly applied for breast cancer therapy, i.e., docetaxel (0–100 nM, Supplement Figure S1A), doxorubicin (0–1,000 nM, Supplement Figure S1B), 5-fluorouracil (0–500 nM, Supplement Figure S1C) or cyclophosphamide (0–1000 µM, Supplement Figure S1D). As a result, the Ki-67 index (Supplement Figure S1, left), a widely used prognostic factor in breast cancer and a marker of proliferation, as well as the relative proliferation rate of the tumor cells in grid-based cell counts (Supplement Figure S1, right panels) decreased in a dose-dependent manner by all tested chemotherapeutics. Of note, their inhibitory potency was not affected by pharmacological blockade of K_Ca_3.1 (Supplement Figure S2), and it did not differ between MMTV-PyMT WT and K_Ca_3.1 KO cells suggesting that the cytotoxic effects of the drugs did not rely on functional K_Ca_3.1 channels.

### 2.2. Irradiation Stimulates K_Ca_3.1 Activity and Ca^2+^ Signals in Breast Cancer Cells 

**Figure 1:**
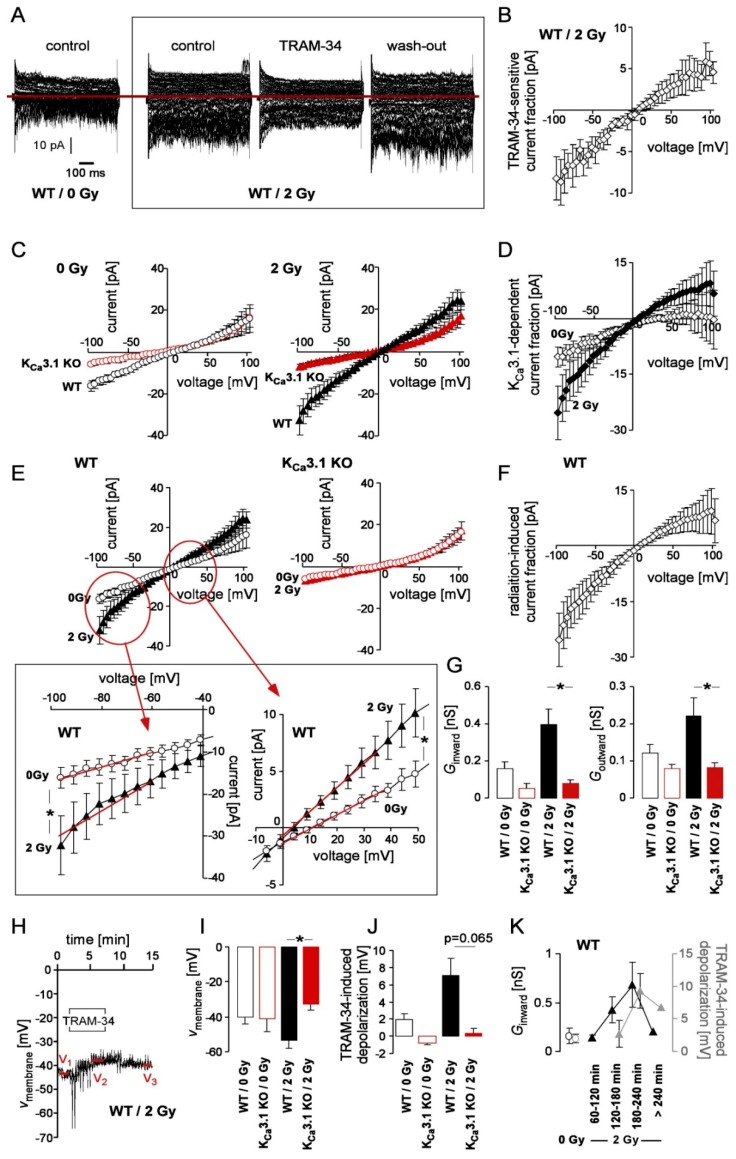
Activation of K_Ca_3.1 channels by irradiation in MMTV-PyMT breast cancer cells. (**A**) Representative macroscopic on-cell current tracings recorded with KCl pipette and NaCl bath solution from an unirradiated (left) and a 2 Gy-irradiated (right) MMTV-PyMT WT cell. Currents of the irradiated cell (right) were obtained before (control), during, and after (wash-out) bath application of TRAM-34 (2 µM). (**B**) Dependence of the mean (±SE, *n* = 5) TRAM-34-inhibited current macroscopic on-cell current fraction on voltage recorded as in (A) in MMTV-PyMT WT cells 180 ± 34 min post-IR with 2 Gy. (**C**) Dependence of the mean (±SE, *n* = 6–20) macroscopic on-cell current fraction on voltage recorded as in (**A**) in unirradiated (open circles, left) and 2 Gy-irradiated (156 ± 12 and 151 ± 6 min post-IR, respectively, closed triangles, right) MMTV-PyMT WT (black) and K_Ca_3.1 KO (red) cells. (**D**) Mean (±SE, *n* = 6–20) K_Ca_3.1-dependent current fraction in unirradiated (open diamonds) and 2 Gy-irradiated (closed diamonds) cells as calculated from the data in (**C**) by subtracting the K_Ca_3.1 currents from those of the WT cells. (**E**) Data of (**C**) replotted to illustrate the IR effect on macroscopic on-cell currents in MMTV-PyMT WT (black, left) and K_Ca_3.1 KO (red, right) cells. The insert below (**E**) shows excerpts of the current-voltage-relationship of unirradiated (open circles) and 2 Gy-irradiated (closed triangle) WT cells in higher power (* indicates *p* ≤ 0.05, two-tailed Welch-corrected *t*-test). (**F**) Mean (±SE, *n* = 11–20) IR (2 Gy)-induced fraction of macroscopic on-cell currents in WT cells as calculated from the data in (**E**) by subtracting currents in unirradiated WT cells from those of the irradiated WT cells. (**G**) Mean (±SE, *n* = 6–20) conductance of the clamped membranes as calculated from the data in (**C**,**E**) for the macroscopic on-cell inward (left) and outward (right) currents in unirradiated (open bars) and 2 Gy-irradiated (closed bars) MMTV-PyMT WT (black) and K_Ca_3.1 KO (red) cells. The voltage ranges used for conductance determination are indicated (in **E**, insert) by the red lines (* indicates *p* ≤ 0.05, Bonferroni-corrected for *n* = 4 pairwise comparisons). (**H**) Time-course of membrane potential (V_membrane_) before during and after (wash-out) application of TRAM-34 as recorded in a 2 Gy-irradiated MMTV-PyMT WT cell in whole-cell current-clamp mode with K-gluconate in the pipette and NaCl in the bath. (**I**) Mean (±SE, *n* = 7–12) membrane potential and (**J**) mean (±SE, *n* = 6–8) TRAM-34-induced membrane depolarization recorded as in (**H**) in unirradiated (open bars) and 2 Gy-irradiated (204 ±14 and 184 ± 15 min post-IR, respectively, closed bars) MMTV-PyMT WT (black) and K_Ca_3.1 KO (red) cells (* indicates *p* ≤ 0.05, Bonferroni-corrected for *n* = 4 pairwise comparisons). (**K**) Time dependence of the IR effect in MMTV-PyMT WT cells as illustrated by changes in membrane potential (black closed triangles) and TRAM-34-induced membrane depolarization (gray closed triangles). For comparison, the corresponding values of the unirradiated WT cells are given (black and gray open circles, respectively). Data are means ± SE with *n* = 3–11 for unirradiated cells and cells recorded 60–240 min post-IR or individual value and mean value(s) (*n* =2) for cells recorded >240 min post-IR.

K_Ca_3.1 reportedly confers radioresistance to glioblastoma cells [21]. Therefore, we assessed K_Ca_3.1 channel activity in control and irradiated (0 or 2 Gy) MMTV-PyMT K_Ca_3.1 WT and KO breast cancer cells. Macroscopic on-cell (cell-attached) currents recorded with KCl pipette solution disclosed a radiation (IR)-induced increase in inward and outward currents (i.e., in the downward and upward deflections of the current tracings, respectively) in K_Ca_3.1 WT breast cancer cells, which were blocked by the K_Ca_3.1 inhibitor TRAM-34 (Figure 1A). The TRAM-34-sensitive current fraction of irradiated WT cells was inwardly rectifying and exhibited a reversal potential close to 0 mV (Figure 1B). Single channel analysis (Supplement Figure S3) suggested a K_Ca_3.1-like inwardly rectifying intermediate conductance (60 pS as calculated for the inward currents) channel with voltage-independent open probability underlying the TRAM-34-sensitive macroscopic current. As a matter of fact, comparing the macroscopic on-cell currents between MMTV-PyMT K_Ca_3.1 WT and KO cells revealed K_Ca_3.1 proficiency-dependent current fractions in unirradiated (Figure 1C, left) and 2 Gy-irradiated (Figure 1C, right) cells that rectified inwardly and increased upon irradiation with 2 Gy (Figure 1D). Notably, the K_Ca_3.1-dependent current fraction in irradiated cells (Figure 1D, closed diamonds) resembled the TRAM-34-sensitive currents (Figure 1C) by rectification behavior and reversal potential albeit being about two-fold larger than the latter.

To analyze the IR effect in both genotypes in more detail, the data of Figure 1C were replotted in Figure 1E to isolate the IR-induced macroscopic current fraction in MMTV-PyMT K_Ca_3.1 WT (left) and KO (right) cells highlighting an IR-induced current only in K_Ca_3.1 WT but not in K_Ca_3.1 KO cells. Not unexpectedly, the radiation-induced current fraction (Figure 1F) resembled the K_Ca_3.1 proficiency-dependent (Figure 1D, closed diamonds) and TRAM-34-sensitive (Figure 1B) current fractions strongly suggesting that irradiation (2 Gy) activates K_Ca_3.1 channels in breast cancer cells. This is also illustrated by comparing the conductances of the clamped membrane between unirradiated and 2 Gy-irradiated MMTV-PyMT K_Ca_3.1 WT and KO cells as calculated for the on-cell inward and outward currents (Figure 1G).

To estimate the functional significance of the IR-induced K_Ca_3.1 activation, the membrane potential was recorded with K-gluconate pipette and NaCl bath solution in the absence and presence of TRAM-34 in fast whole-cell mode in unirradiated and 2 Gy-irradiated MMTV-PyMT K_Ca_3.1 WT and KO cells (Figure 1H). As a result, the membrane potential under all 4 experimental conditions was about 35–45 mV more positive than the K^+^ electrochemical equilibrium potential (−88 mV) indicating significant contributions of non-K^+^-selective ion channels to the membrane potential in these cells (Figure 1I). Irradiation induced a (not significant) hyperpolarization of the membrane potential (Figure 1I) in MMTV-PyMT K_Ca_3.1 WT cells but not in KO cells. Importantly, upon irradiation (but not under control conditions) the membrane potential of WT cells was significantly more negative than that of KO cells (Figure 1I). This difference was paralleled by a TRAM-34-mediated membrane depolarization. In particular, TRAM-34 depolarized the membrane potential in unirradiated (*p* = 0.074, one-sample t-test against 0, Bonferroni-corrected for *n* = 4 tests) and 2 Gy-irradiated KCa3.1 WT cells (*p* = 0.055, *n* = 4 tests) in an almost significant manner. Pooling the unirradiated and 2 Gy-irradiated data indicated a highly significant TRAM-34-mediated depolarization of the membrane potential in K_Ca_3.1 WT (*p* = 0.005, *n* = 2 tests) but not in in the pooled K_Ca_3.1 KO cells (*p* = 0.8, *n* = 2 tests, data not shown). As a result, and as indicated in Figure 1J, the TRAM-34-mediated depolarization of the membrane potential differed almost significantly (p = 0.065, *n* = 4 pairwise comparison) between irradiated WT and K_Ca_3.1 KO cells (Figure 1J). Since no IR or TRAM-34 effect on membrane potential was apparent in K_Ca_3.1 KO cells (Figure 1I,J) this strongly suggests that IR-induced KCa3.1 channel activation may impact on the membrane potential of breast cancer cells. In our experiments, a transient K_Ca_3.1-mediated hyperpolarization of the membrane potential developed with a time lag of 2–3 h and declined >4 h post-IR (Figure 1K).

**Figure 2:**
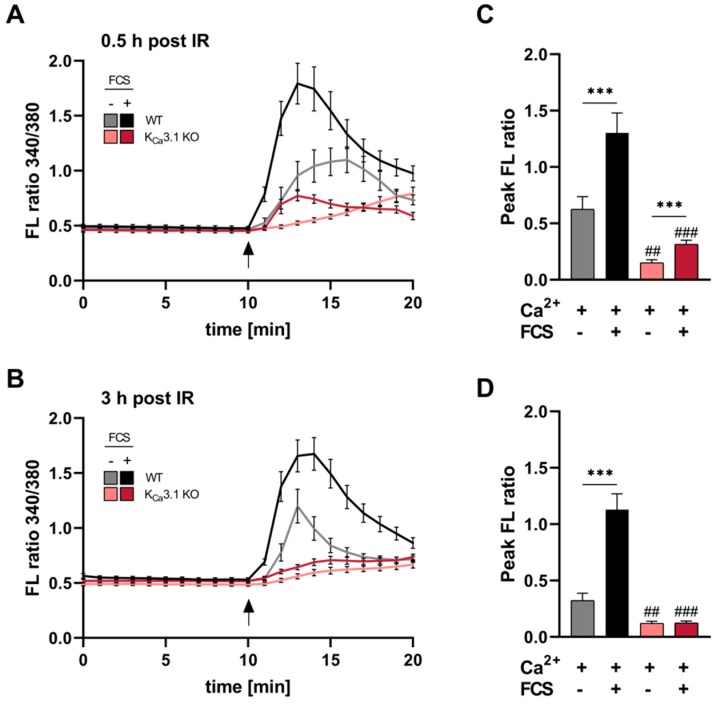
IR-induced [Ca^2+^]_i_ signals in MMTV-PyMT breast tumor cells depend on K_Ca_3.1 channels (**A,B**) Acute Ca^2+^ signals of MMTV-PyMT mammary tumor cells evoked by Ca^2+^ (1.8 mM) superfusion only (gray and light red lines) or Ca^2+^ (1.8 mM) plus 5% fetal calf serum (FCS; black and red lines) after 30 or 180 min of irradiation (2 Gy). Irradiated FURA-2-AM-loaded cells were monitored in Ca^2+^-free buffer for 10 min prior to the respective treatments as indicated. Arrow indicated buffer replacement. (**C,D**) The mean peak fluorescence (FL) ratio of the 340/380 nm wavelength was determined relatively to the respective baseline FL ratio for MMTV-PyMT K_Ca_3.1 KO (*n* = 14–28 cells per condition derived from three different breast tumors) and WT (*n* = 21–27 cells per condition derived from three different breast tumors) groups. Statistical analysis was performed by two-way analysis of variance (ANOVA) with * *p* < 0.05, ** *p* < 0.01, *** *p* < 0.001 indicating statistical differences between FCS-treated and -untreated groups and ^##^ *p* < 0.01, ^###^ *p* < 0.001 for differences between genotypes either at Ca^2+^ plus FCS or Ca^2+^ “only” condition.

Following the IR-induced activation of K_Ca_3.1 currents (Figure 1), we studied whether IR contributed to intracellular Ca^2^ ([Ca^2+^]_i_) signaling in an K_Ca_3.1-dependent manner. In earlier studies, we observed that growth factor-dependent changes in [Ca^2+^]_i_ homeostasis required K_Ca_3.1 [10], and IR as well as Ca^2+^, in turn, may further activate K_Ca_3.1 in MMTV-PyMT breast cancer leading to a sustained entry of Ca^2+^ along its electrochemical gradient through e.g., Orai/STIM channels [22,23]. Hence, regulatory effects of K_Ca_3.1 on Ca^2+^ dynamics were determined in Ca^2+^- and growth factor exposed un-/irradiated MMTV-PyMT K_Ca_3.1 WT and KO breast cancer cells (Figure 2A,C). In comparison to unirradiated cells (Supplement Figure S4), Ca^2+^ superfusion as well as co-treatment with growth-factors (i.e., fetal calf serum (FCS)) resulted in a robust IR-induced [Ca^2+^]_i_ signal in MMTV-PyMT K_Ca_3.1 WT cells. In line with previous Ca^2+^ recordings [10], Ca^2+-^ and FCS-induced [Ca^2+^]_i_ signal gradients and amplitudes were significantly lower in the absence of K_Ca_3.1 (Supplement Figure S4A,B). Moreover, mean peak FL ratios of IR_30_-exposed (Figure 2C) versus IR_sham_ treated (Supplement Figure S4B) K_Ca_3.1 WT cells differed by +0.26 (Ca^2+^ “only”) and +0.69 (Ca^2+^ plus FCS), whereas the respective values obtained from K_Ca_3.1 KO cells (+0.08 for Ca^2+^ “only” and −0.04 for Ca^2+^ plus FCS) imply that IR affects [Ca^2+^]_i_ through a K_Ca_3.1-dependent mode. Accordingly, peak [Ca^2+^]_i_ amplitude after irradiation of FCS-treated and -untreated K_Ca_3.1-deficient cells only differed marginally at IR_30_ min (Figure 2C), and even this effect was lost after IR_180_ min (Figure 2D). Together, these findings strongly suggest that loss of K_Ca_3.1 largely abrogate early and late post-irradiation [Ca^2+^]_i_ signals essential for tumor cell functions such as cell migration, proliferation, invasion and survival [24].

### 2.3. Clonogenic Survival of Irradiated Breast Cancer Cells Depends on K_Ca_3.1 Function

**Figure 3:**
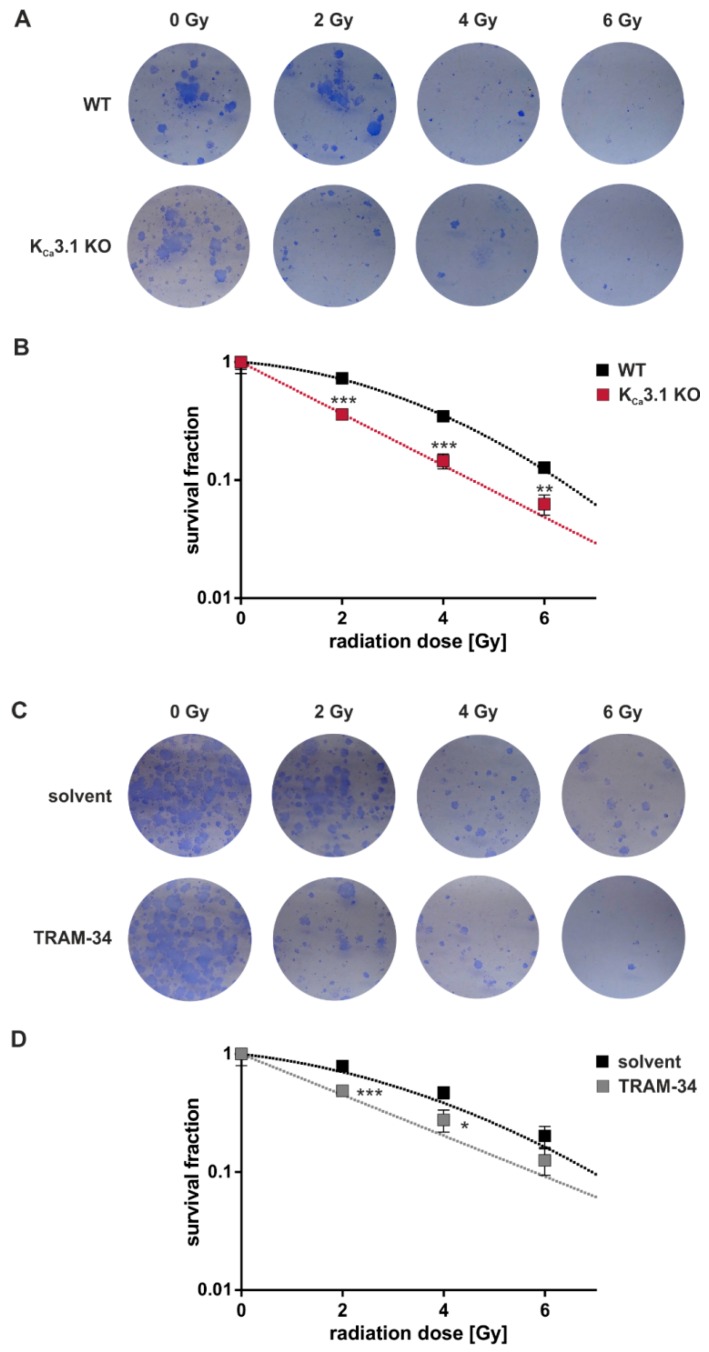
Clonogenic survival of irradiated MMTV-PyMT breast cancer cells in vitro depends on K_Ca_3.1. (**A**) Light micrographs of colonies formed from MMTV-PyMT WT (upper row) and K_Ca_3.1 KO (lower row) cells upon exposure to 0, 2, 4, or 6 Gy ionizing radiation. (**B**) Mean (±SE, *n* = 9 with 6 technical replicates per experiment) survival fractions of irradiated (0–6 Gy) K_Ca_3.1 WT (black) and KO cells (red) MMTV-PyMT breast cancer cells as determined as in (**A**) by delayed plating colony formation assay with data given semi-logarithmically as survival fraction/radiation dose plot. Survival fractions are given in semi-logarithmic plots because otherwise differences at higher radiation doses cannot be resolved easily by linear plots. (**C**) Light micrographs of colonies formed from MMTV-PyMT WT cells upon exposure to 0, 2, 4, or 6 Gy ionizing radiation. Radiation and 24 h post-incubation were performed in the absence (solvent) or presence of TRAM-34 (10 µM). (**D**) Mean (±SE, *n* = 5 with 6 technical replicates per experiment) survival fractions of irradiated (0–6 Gy) WT MMTV-PyMT breast cancer cells co-treated with solvent (black) or TRAM-34 (red) as determined as in (**C**) by delayed plating colony formation assay. Data are given semi-logarithmically as survival fraction/radiation dose plot. (**B**,**D**) *, ** and *** indicate *p* ≤ 0.05, *p* ≤ 0.01 and *p* ≤ 0.001, respectively, in unpaired *t*-tests.

Delayed plating colony formation assays were performed to determine K_Ca_3.1 channel-dependent clonogenic survival after irradiation. The number of colonies as defined by clusters of ≥ 50 cells were divided by the number of plated cells to obtain the plating efficiency. Survival fractions of the irradiated cells in each experimental group were calculated by normalizing the plating efficiencies to that of the respective 0 Gy control group (Figure 3). The survival fraction curves of the untreated WT cells (Figure 3B,D) showed an upward deflecting “shoulder” which follows a linear quadratic function. This deflection represents the capacity of the WT cells to repair radiation-induced DNA double-strand breaks al lower radiation doses. Importantly, K_Ca_3.1 deficiency and TRAM-34 flattened the curve (Figure 3B,D) pointing to the K_Ca_3.1-dependency of DNA repair. In summary, genetic KO (Figure 3A,B) and pharmacological blockade (Figure 3C,D) of K_Ca_3.1 decreased survival fractions of the irradiated cells as compared to WT cells and vehicle control, respectively, indicating a radioresistance-conferring function of K_Ca_3.1.

### 2.4. K_Ca_3.1 Channels are Required for the Radiation-Induced DNA Damage Response

**Figure 4:**
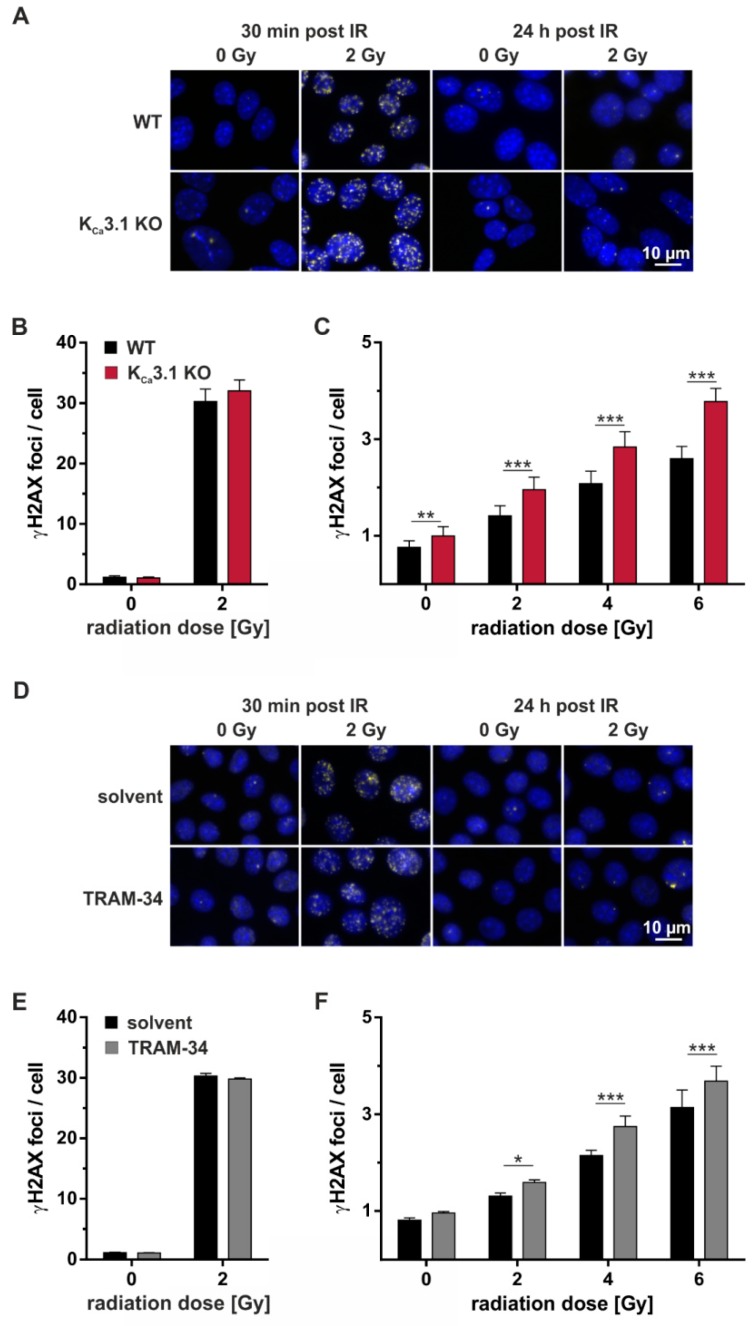
K_Ca_3.1 channels promote repair of DNA double-strand breaks. (**A**) Fluorescence micrographs showing nuclear γH2AX foci as a measure of DNA double-strand breaks 30 min (left) and 24 h (right) after irradiation of MMTV-PyMT WT (upper row) and K_Ca_3.1 KO cells (lower row) with 0 Gy or 2 Gy. (**B,C**) Mean number (±SE, *n* = 7) of nuclear γH2AX foci in MMTV-PyMT WT (black) and K_Ca_3.1 KO (red) cells (**B**) 30 min and (**C**) 24 h after irradiation. (**D**) Fluorescence micrographs showing nuclear γH2AX foci 30 min (left) and 24 h (right) after irradiation of MMTV-PyMT WT with 0 Gy or 2 Gy. Irradiation and 24 h post-incubation were either performed in the absence (solvent, upper row) or presence (lower row) of TRAM-34 (10 µM). (**E**,**F**) Mean number (±SE, *n* = 5) of nuclear γH2AX foci in MMTV-PyMT WT cells (**E**) 30 min and (**F**) 24 h (red) after irradiation and post-incubation in the absence (black bars) or presence (gray bars) of TRAM-34 (10 µM). γH2AX foci in (**A**,**D**) are shown in yellow, DAPI-stained nuclei appear in blue. *, **, and *** indicate *p* ≤ 0.05, *p* ≤ 0.01, and *p* ≤ 0.001, respectively, two-way ANOVA with Sidak’s post-hoc test.

To detect radiation-induced nuclear DNA double-strand breaks, γH2AX foci were analyzed by immunofluorescence microscopy. Thirty min after irradiation with 0 or 2 Gy, numbers of radiation-induced DNA double-strand breaks were modified by neither KO (Figure 4A, left, and Figure 4B) nor by pharmacological blockade (Figure 4D, left, and Figure 4E) of K_Ca_3.1 indicative of radiation-induced DNA double-strand break formation that was independent from K_Ca_3.1. In contrast, numbers of residual nuclear γH2AX foci after 24 h of DNA repair were higher in K_Ca_3.1-deficient than in -proficient cells (Figure 4A, right, and Figure 4C) and in TRAM-34-treated than vehicle-incubated K_Ca_3.1 WT cells indicating a function of K_Ca_3.1 in the DNA damage response.

### 2.5. High Vulnerability of K_Ca_3.1-Deficient Breast Cancer cell-*Derived Tumor Grafts* to Ionizing Radiation 

**Figure 5:**
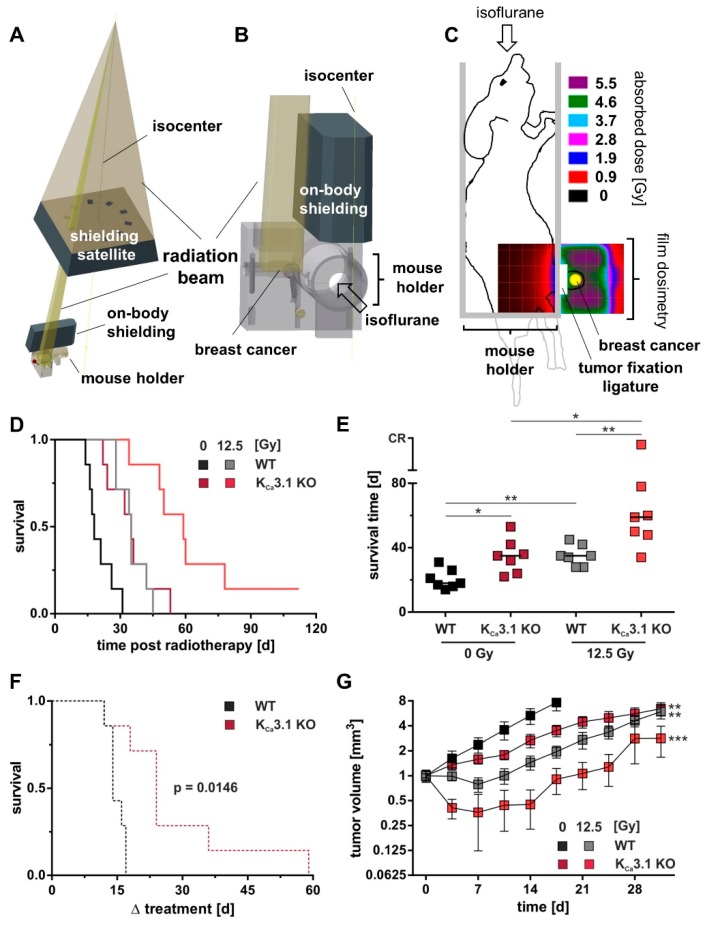
K_Ca_3.1 channels confer radioresistance to MMTV-PyMT breast cancer in vivo. Computer-aided depiction of the irradiation setup used to locally irradiate the breast tumors. Part (**A**) represents the radiation beam (yellow gold-colored) produced by a linear accelerator and the lead shieldings. The beam is trimmed by the shielding satellite to a rectangular miniature beam which then reaches the tumor. (**B**) Beam, mouse holder, and 2nd on-body shielding in higher magnification. (**C**) Film dosimetry recorded in the coronal plane of a mouse phantom during application of 5 Gy with the setup described in A,B. Drawings of mouse and holder are superimposed. Dose distribution is given in pseudo colors. (**D**) Kaplan-Meier curves and (**E**) individual (symbols) and median (line) survival time (*n* = 7) of FVB/N mice orthotopically transplanted with MMTV-PyMT WT (black/gray) or K_Ca_3.1 KO (red) cells and treated with 5 fractions for a total dose of either 0 Gy (black, dark red) or 12.5 Gy (gray, bright red) starting at a tumor size of 5 mm. Compared to WT 0 Gy or 12.5 Gy, survival time was significantly prolonged in accordingly treated mice bearing K_Ca_3.1 KO tumors with one complete responder (CR) in the ionizing radiation group. *, **, and *** indicate *p* ≤ 0.05, *p* ≤ 0.01 and *p* ≤ 0.001. Survival statistics in (**E**) are calculated from Kaplan-Meier curves in (**D**) by log-rank test with Bonferroni correction. (**F**) Kaplan-Meier curves showing radiation-mediated survival benefit for both genotypes as calculated by survival_benefit_ = 1 − (survival_12.5Gy_ − survival_0Gy_). Normalized to non-irradiated controls was performed to present in detail the survival benefit of the respective genotypes from IR. The indicated p value was calculated by log-rank test. (**G**) Mean tumor volume (±SE, *n* = 7) normalized for each mouse to the volume at treatment start is plotted against the time for control (0 Gy, gray and bright red) and 12.5 Gy-fractionated irradiated (black and dark red) K_Ca_3.1 WT (gray, black) and KO (bright and dark red) tumors. Two-way ANOVA and Tukey´s test compared to WT 0 Gy with ** and *** indicative for *p* ≤ 0.01 and *p* ≤ 0.001.

To test whether tumor K_Ca_3.1 deficiency may also be associated with radiosensitivity of MMTV-PyMT breast tumors in vivo, K_Ca_3.1-proficient or -deficient cells were transplanted orthotopically into syngeneic K_Ca_3.1 WT mice. At a size of about 5 mm tumors were treated with either a total dose of 0 or 12.5 Gy delivered in five fractions (Figure 5A–C) at five consecutive days. As a result, K_Ca_3.1 KO breast tumors progressed slower than WT tumors after tumor cell inoculation (Figure 5D,E). Accordingly, untreated mice challenged with K_Ca_3.1 KO cells (35 days) showed a significant longer median survival time as compared to those with K_Ca_3.1 WT tumors (18 d; Figure 5D,E, black and dark red lines/symbols). Importantly, radiation (12.5 Gy in 5 fractions) prolonged median survival time to a larger extent (24 days) in mice bearing K_Ca_3.1 KO tumors (Figure 5D,E, dark red and bright red lines/symbols) than in mice with WT lesions (17 days; Figure 5D,E, black and gray lines/symbols). This is also illustrated in the Kaplan-Meier curves confirming a radiation-mediated survival benefit for mice challenged with K_Ca_3.1 KO WT (black) and KO (red) tumors (Figure 5F). Given the fact that the median volume at radiation begin did not differ between irradiation versus non-irradiation groups (43 mm³ versus 46 mm³ for WT and 86 mm³ versus 67 mm³ for K_Ca_3.1 KO), this higher radiation-induced extension of mean survival in mice with K_Ca_3.1 KO tumors (Figure 5F) is best explained by a clonogenic survival that was lower in irradiated K_Ca_3.1 KO tumors than in WT tumors. This is also evident from the radiation-mediated delay in tumor volume increase, which was larger for K_Ca_3.1 KO than for WT tumors (Figure 5G.) In addition, such in vivo radioprotective function of K_Ca_3.1 can be deduced from the observation that mice with irradiated K_Ca_3.1 KO tumors showed the highest scatter towards longer survival times with one complete therapy responder (Figure 5E). Collectively, these in vivo findings imply that K_Ca_3.1 promotes breast cancer development and radioresistance in the MMTV-PyMT mouse model.

## 3. Discussion

K_Ca_3.1 channels may interfere with resistance to chemotherapy of tumors as suggested by few studies showing that K_Ca_3.1 function is required in human epidermoid cancer cells [14] and glioblastoma cells [25] for caspase activation following treatment with cisplatin and staurosporine, respectively. In particular, K_Ca_3.1 was needed for apoptotic cell volume decrease as a prerequisite of caspase activation. Contrary to this pro-apoptotic function, K_Ca_3.1 channels were reported to confer resistance to TMZ in glioblastoma [16]. Combined, these data might hint to a complex interaction of K_Ca_3.1 with stress response pathways of tumor cells. A further study, however, which associated K_Ca_3.1 abundance and sensitivity to staurosporine, C2-ceramide, and cisplatin-induced cell death in a large panel of tumor cell lines, did not find a correlation between K_Ca_3.1 abundance and drug sensitivity [26]. In the present study, K_Ca_3.1 KO had no effect on the sensitivity of MMTV-PyMT breast cancer cells to docetaxel, doxorubicin, 5-fluorouracil, or cyclophosphamide. One might conclude from these observations that interference of K_Ca_3.1 function with the drug-triggered stress response does not seem to be a widespread phenomenon in tumor biology [26].

Unlike its incompletely understood role in chemotherapy, K_Ca_3.1 has consistently been demonstrated to exert pro-survival functions in ionizing radiation-treated T cell leukemia [27], lung adenocarcinoma [19], and glioblastoma [21,28]. Among these functions is the maintenance of up-regulated sodium-coupled glucose fueling [29] during the DNA damage response. Besides energy for the highly ATP-demanding stress response, accelerated glucose uptake reportedly also provides substrates for histone acetylation as a prerequisite for DNA decondensation [30].

Upstream of Ca^2+^-activated K^+^ channels, IR has been reported in glioblastoma cells to induced Ca^2+^ store release via stabilization of hypoxia-inducible factor 1α and up-regulation of auto-/paracrine chemokine signaling [31] as well as in glioblastoma and leukemia cells Ca^2+^ entry via yet ill-defined activation of members of the transient receptor potential (TRP) family of Ca^2+^-permeable nonselective cation channels [27,31]. Beyond K^+^ transport, K_Ca_3.1 probably in concert with these TRP channels and/or L type Ca^2+^ channels contributes to Ca^2+^ signaling [18,27,32,33]. Through hyperpolarizing the membrane, K_Ca_3.1 channels modify passive Ca^2+^ entry in unexcitable cancer cells, and this influx may in turn generate a positive feedback loop on K_Ca_3.1 activity [2]. As a matter of fact, IR-induced activity of Ca^2+^-activated K_Ca_3.1 and/or BK_Ca_ K^+^ channels [34] has been demonstrated to contribute to Ca^2+^ signaling required to activate Ca^2+^ effector proteins involved in cell cycle arrest during the DNA damage response [18]. In particular, radiation-induced Ca^2+^ signals in glioblastoma and leukemia cells were shown to activate isoforms of the Ca^2+^/calmodulin-dependent kinase II (CaMKII), which via inactivating phosphorylation of cdc25 phosphatases keeps the cdc2 subunit of the mitose-promoting factor in a phosphorylated and thus inactive form resulting in G_2_/M cell cycle arrest. Accordingly, knockdown or inhibition of K_Ca_3.1 compromises G_2_/M cell cycle arrest that may result in mitotic catastrophe of DNA-damaged cells via premature entry into mitosis [21]. Similarly, in the present study, we find acute as well as sustained post-irradiation [Ca^2+^]_i_ fluctuations to require functional K_Ca_3.1 channels in MMTV-PyMT breast cancer cells. In glioblastoma cells, a dependence of radiation-triggered stress response on K_Ca_3.1 function is illustrated by the radiosensitizing effect of TRAM-34 [21]. In addition to overriding G_2_/M cell cycle arrest, TRAM-34 delays repair of DNA double-strand breaks in these cells as disclosed by an increased number of residual γH2AX foci [21] pointing to a further role of K_Ca_3.1 in DNA repair. In the present study, ionizing radiation stimulated the activity of K_Ca_3.1 in MMTV-PyMT breast cancer cells similar to the situation in leukemia [27], adenocarcinoma [19] and glioblastoma cells [21]. Furthermore, TRAM-34 or K_Ca_3.1 KO radiosensitzed MMTV-PyMT cells in vitro. The corresponding decline in clonogenic survival was accompanied by a delayed repair of DNA double-strand breaks of MMTV-PyMT cells in vitro pointing to an involvement of K_Ca_3.1 in DNA damage response and DNA double-strand repair.

In our previous study, K_Ca_3.1 deficiency did not decrease the incidence of breast cancer formation and tumor growth in the spontaneous MMTV-PyMT mouse model [10] suggesting an independence from neoplastic transformation regarding K_Ca_3.1 function. In the present study, K_Ca_3.1 KO tumors progressed slower than WT tumors when transplanted orthotopically into syngeneic WT mice. This was also associated with a prolonged median survival of K_Ca_3.1 KO as compared to WT breast tumor-bearing mice challenged with K_Ca_3.1 WT tumors. Notably, fractionated irradiation of such tumors prolonged survival of K_Ca_3.1 KO more than that of WT tumor-bearing mice indicative of a radioprotective function of K_Ca_3.1 also in the in vivo situation. These effects were attributed to K_Ca_3.1 channels localized at the plasma membrane, yet K_Ca_3.1 is also known to be present in mitochondria and our present work does not exclude an additional contribution of this intracellular population of K_Ca_3.1 channels to the IR-associated tumor cell behaviors [35].

Beyond its function in the stress response during radiotherapy, K_Ca_3.1 is up-regulated in mesenchymal glioblastoma stem cells [28]. Of note, the highly migratory phenotype of glioblastoma stem cells depends on K_Ca_3.1 function [36]. Likewise, in triple-negative breast cancer cells, epithelial-mesenchymal transition (EMT) is associated with up-regulation of K_Ca_3.1 channels and cell migration requires functional K_Ca_3.1 channels [37]. Since EMT was shown to precede tissue invasion and distant metastasis of carcinoma [38,39] including breast cancer [40], it is tempting to speculate that K_Ca_3.1 promotes distant metastasis of breast cancer. Of note, ionizing radiation was demonstrated to stimulate migration of glioblastoma cells [21,31] by triggering signaling cascades that involve K_Ca_3.1 [20]. The similarities between K_Ca_3.1 channel function in glioblastoma and breast cancer might give rise to the speculation that K_Ca_3.1 also promotes metastasis of irradiated breast cancer. Along those lines, local tumor irradiation in a syngeneic mouse model of orthotopic breast cancer was shown to promote distant lung metastasis [41].

## 4. Material and Methods

### 4.1. MMTV-PyMT Murine Breast Cancer Primary Cell Culture

MMTV-PyMT WT and K_Ca_3.1 KO female mice [10,42,43] were backcrossed on FVB/N background that develop spontaneously breast tumors [44]. The MMTV-PyMT mouse line is widely employed in breast cancer research. Comparable to the stages and morphology of human breast cancer, MMTV-PyMT mice develop hyperplasia followed by adenoma, and early and late carcinoma. Stage-dependent overexpression (or loss) of prominent breast cancer biomarkers including estrogen and progesterone receptors, cNeu/HER2 and/or cyclin D1 are characteristic for MMTV-PyMT tumors [45,46]. The genetic metastasis signature of MMTV-PyMT as well as the ability of primary tumors to spread to the lung further mimic important features of human breast cancer [44,47]. Primary cell cultures from this model were established from excised tumors as previously described [10]. Tumor cells were grown at 37 °C in a 5% CO_2_ and H_2_O-saturated atmosphere in improved minimal essential medium (IMEM) medium supplemented with 0 or 5% FCS and 1% penicillin-streptomycin (all Thermo Fisher Scientific, Waltham, MA, USA). Media were replaced twice per week.

### 4.2. Syngeneic Orthotopic MMTV-PyMT Transplant Model and Radiotherapy

MMTV-PyMT K_Ca_3.1 WT or KO cells were transplanted in the fourth right mammary gland of 12-week-old female FVB/N WT mice (Charles River, Sulzfeld, Germany). For surgery, animals were anaesthetized by intraperitoneal injection of 100 mg/kg body weight of ketamine (Inresa, Freiburg, Germany) and 10 mg/kg body weight of xylazine (Bernburg, Bernburg, Germany). With the mouse placed on a heating pad, the mammary gland was shaved and disinfected. Between the ventral midline and the fourth mammary gland, an approximate 10 mm caudocranial cut was made to expose the mammary fat pad. Tumor cells (10^6^ in 50 µL PBS) were injected into the mammary gland and the wound margins tightly closed using a 6-0 suture. Post-operative pain was controlled by metamizole (200 mg/kg body weight, 1 A Pharma, Oberhaching, Germany). Inoculated mice were palpated twice per week for tumor onset. Body weight, motility, grooming, and other mouse health criteria were routinely monitored. Tumor volume was calculated according to the formula (0.5 length × width²). To define the radioresistance of orthotopic K_Ca_3.1 WT MMTV-PyMT breast tumors, mice with tumor diameters of around 5 mm (62.5 mm³) were allocated to four groups. Tumors were irradiated under isoflurane anesthesia with a total dose of 0, 12.5, 25 or 35 Gy 6 MV photons in five fractions using of a linear accelerator (LINAC SL25, Philips, Figure 5A–C, Supplement Figure S5A,B). During irradiation, the body of the mice was protected by two lead shielding blocks (Figure 5A,B) with the breast tumor pulled aside outside the shielded area and gently fixed with a ligature (Figure 5C). Dosimetry was performed by irradiation of Gafchromic 3 films (Ashland Inc., Covington, LA, USA) placed in a mouse phantom (Figure 5C). Here, dose distribution (5 Gy) is given in pseudo colors. Tumor volume and survival time (i.e., the time until the tumor volume reached an eight-fold increase compared to the first day of irradiation) were monitored. The initially performed fractionated irradiation of orthotopic K_Ca_3.1 WT tumors with 0, 12.5, 25 or 35 Gy total dose indicated a significant increase in survival time and delay in tumor growth already at 12.5 Gy (5 × 2.5 Gy) as compared to the mock-irradiated (0 Gy) group (Supplement Figure S5A,B). In addition, this treatment protocol was well tolerated by the mice as deduced from the body weight increase during fractionated radiation that did not differ between the four groups (Supplement Figure S5C,D). Therefore, this fractionation scheme was chosen for a further series of experiments to define the function of K_Ca_3.1 in the radioresistance of MMTV-PyMT breast tumors by comparing WT and K_Ca_3.1 KO tumors grown orthotopically in WT mice. All animal experiments were approved by the local Ethics Committee for Animal Research (Regierungspraesidium Tuebingen, No. PZ2/10, PZ1/16 and PZ2/17).

### 4.3. K_Ca_3.1 Inhibition and Cytotoxic Drugs

TRAM-34 (Alomone, Jerusalem, Israel) was dissolved in ethanol (Sigma Aldrich, Taufkirchen, Germany) at a stock solution of 5 mM and further diluted to final concentrations of 2 µM for patch-clamp recording and 10 µM for clonogenic survival or γH2AX assays. Chemotherapeutics were prepared in dimethyl sulfoxide (DMSO) at stock concentrations of 5 mM (5-fluorouracil), 10 mM (docetaxel and doxorubicin), or 50 mM (cyclophosphamide; all chemicals from Sigma Aldrich, Taufkirchen, Germany) and applied after serial dilution.

### 4.4. Ki-67 Proliferation Analysis

Eight-well chamber slides (Sarstedt, Sarstedt, Germany) were used to seed 50,000 MMTV-PyMT WT or K_Ca_3.1 KO cells per well in duplicate. After 24 h of culture in 5% FCS-containing medium and subsequent serum starvation for 72 h, cells were treated with increasing concentrations of docetaxel, doxorubicin, 5-fluoruracil or cyclophosphamide for further 72 h. Then, cells were fixed with 70% ethanol at −20°C for 10 min, washed three times with PBS (Thermo Fisher Scientific) and incubated in 0.1% TritonX100 (Carl Roth, Karlsruhe, Germany) in PBS for 15 min. Cells were gently washed another three times with PBS before 10% normal donkey serum (Dianova, Hamburg, Germany) in PBS was applied for 1 h to block unspecific epitopes. After removing the blocking solution, the cells were incubated in an anti-Ki-67 rabbit primary antibody (9129S, Cell Signaling Technology, Frankfurt am Main, Germany) diluted 1:1,000 in 1.5% normal donkey serum in PBS for 2 h. After three washing steps in PBS, cells were incubated for 1 h with 1:800-diluted Alexa Fluor^®^ 555 donkey anti-rabbit secondary antibody (A31572, Thermo Fisher Scientific) solution, which was prepared in 1.5% normal donkey serum in PBS. Finally, cells were washed another three times in PBS and embedded under a cover slip with DAPI-containing vectashield antifade mounting medium (Vector Laboratories, Lorrach, Germany). Four independent areas from each chamber were considered for evaluating Ki-67-positive and -negative cells and the resulting ratio is referred to as Ki-67 index.

### 4.5. Cell Count

MMTV-PyMT WT or K_Ca_3.1 KO cells (80,000) were seeded in duplicate to adhere for 24 h in grid-500 µ-dishes (ibidi, Planegg, Germany). After 72 h of serum withdrawal, cells were restimulated with 5% FCS-containing phenol red-free cell growth medium with addition of 100 nM docetaxel, 1000 nM doxorubicin, 500 nM 5-fluorouracil, 1000 µM cyclophosphamide or control conditions. Digital images of previously defined areas were captured every 24 h for in total 72 h and cell counts were determined by use of the ImageJ software (Version 1.52a, Wayne Rasband, National Institute of Health, Bethesda, MD, USA).

### 4.6. Patch-Clamp Recording 

Suspensions of 40,000 to 80,000 MMTV-PyMT WT or K_Ca_3.1 KO cells were grown for 4 to 7 days in 3 cm culture dishes (Corning, Amsterdam, Netherlands). Prior to patch-clamp analysis, K_Ca_3.1 KO or WT breast tumor cells were irradiated with a single fraction of 0 or 2 Gy. Recording was performed in on-cell (cell-attached) mode 60–320 min after irradiation. Currents were evoked by 41 voltage square pulses (700 ms each) from 4 mV holding potential to voltages between −96 mV and +104 mV delivered in 5 mV increments. Voltages were corrected off-line for the estimated liquid junction potential and refer to the cytoplasmatic face of the plasma membrane in respect to the earthed extracellular face and were applied on top of the physiological membrane potential of the cell. Cells were superfused at 37 °C with NaCl solution (in mM: 125 NaCl, 32 n-2-hydroxyethylpiperazine-n-2-ethanesulfonic acid (HEPES), 5 KCl, 5 d-glucose, 1 MgCl_2_, 2.5 CaCl_2_, titrated with NaOH to pH 7.4). The pipette solution contained (in mM) 130 KCl, 32 HEPES, 5 d-glucose, 1 MgCl_2_, 1 CaCl_2_, titrated with KOH to pH 7.4. In some experiments, the K_Ca_3.1 inhibitor TRAM-34 (2 µM in bath solution) was applied. For single channel analysis, on-cell currents (KCl pipette and NaCl bath solution) were sampled for longer time periods at voltages between −100 mV and +100 mV in 10 mV increments.

Membrane potential was recorded at 37 °C in fast whole-cell, current-clamp mode. After entry into the whole-cell mode the current was clamped to 0 pA and the membrane voltage continuously recorded with a pipette solution containing (in mM): 140 K-D-gluconate, 5 HEPES, 5 MgCl_2_, 1 K-ethylene glycol-bis(-aminoethyl ether)-N,N,N′,N′-tetraacetic acid, (EGTA), 1 K-ATP, titrated with KOH to pH 7.4. Records were obtained during constant superfusion with the NaCl solution (see above) during wash-in and wash-out of TRAM-34 (1 µM in bath solution). Voltages (V) were analyzed by averaging the values over a time period of 40–60 ms directly before blocker wash-in (V_1_, see Figure 1H, at the end of TRAM-34 application (V_2_) and at the end of the wash-out period (V_3_). TRAM-34-induced voltage changes (ΔV) were calculated by the following quotation:

ΔV = ((V1 − V2) + (V3 − V2))/2

### 4.7. Calcium Imaging

Intracellular calcium [Ca^2+^]_i_ of murine breast tumor cells was determined with chelating agent FURA-2-AM (Merck Millipore, Darmstadt). The two different excitation-wavelengths of FURA-2-AM were used to ascertain their fluorescence emission ratio (FL 340/380), which correlates with the Ca^2+^ bound (340 nm) and Ca^2+^ free (380 nm) signals. 80,000 cells were plated, pre-treated and loaded as described previously [10]. Upon FURA-2-AM loading cells remained either unirradiated or were irradiated using 2 Gy of IR in one cycle. Cells were washed in 2 ml Ca^2+^-free incubation buffer to dispose remaining FURA-2-AM prior to investigating [Ca^2+^]_i_, for a period of 20 min using VisiView (Visitron) and Spot Inside camera (Visitron) attached to an inverse microscope (Axiovert S100, halogene lamp XBO 75, Carl Zeiss, Jena). Every 2 seconds, digital pictures were taken at 340 and 380 nm. After baseline recording in Ca^2+^-free buffer, cells were superfused for 10 min with buffer containing either 1.8 mM Ca^2+^ or 1.8 mM Ca^2+^ plus 5% fetal calf serum (FCS). Fluorescence intensity of the single cells of every picture sequence was determined with ImageJ software.

### 4.8. Colony Formation Assay

Clonogenic survival after irradiation was assessed 72 h after seeding of 600,000 MMTV-PyMT WT or K_Ca_3.1 KO cells in 25 cm² cell culture flasks (Corning, Amsterdam, Netherlands). In further experiments, MMTV-PyMT WT cells were treated with 10 µM TRAM-34 or ethanol used as a solvent 1 h prior to irradiation. Then, cells of the different genotypes or treatments were irradiated with radiation doses of 0, 2, 4, or 6 Gy. After 24 h, delayed plating in six-well plates with six technical replicates of 3500 cells per well was performed (Corning, Amsterdam, The Netherlands). Colonies were defined by clusters of at least 50 cells. Subsequent to 14 days of culture, colonies were fixed with 3.7% formaldehyde (Merck, Darmstadt, Germany) in PBS for 10 min. For colony staining, formaldehyde solution was replaced by 70% ethanol for 10 min, cells were shortly washed with H_2_O twice and finally incubated with 0.05% coomassie solution for 10 min until staining of individual colonies was macroscopically visible. After removal of the staining solution, wells were rinsed with H_2_O before six-well plates were air-dried and the colonies in each well were counted manually under a microscope.

### 4.9. γH2AX Foci Analysis

For the analysis of formation and repair of DNA double-strand breaks during and after irradiation, 50,000 MMTV-PyMT WT or K_Ca_3.1 KO cells were seeded for 72 h in twelve-well chambers (ibidi, Planegg, Germany) in triplicate. TRAM-34 or its solvent was applied 1 h prior to irradiation with a single fraction of 0, 2, 4, or 6 Gy. After further 30 min or 24 h of culture, cells were fixed with 70% ethanol at −20°C for 10 min. Then, the cells were washed twice with 250 µL PBS and unspecific epitopes were blocked with 10% normal goat serum (Linaris, Dossenheim, Germany) in PBS for 1 h. Blocking solution was removed and the cells were incubated for 2 h in a primary mouse anti-γH2AX antibody (613402, BioLegend, London, UK) solution (1:500 dilution in 1.5% normal goat serum/PBS). Next, cells were washed twice with PBS and incubated with a secondary Alexa Fluor^®^ 555 goat anti-mouse antibody (A21127, Thermo Fisher Scientific) diluted 1:800 in 1.5% normal goat serum in PBS for 1 h. After two final PBS washing steps, the silicone chamber walls were removed, and the slide was cover-slipped with DAPI-containing vectashield solution. Four micrographs of different areas were taken using the F-view II camera (SIS, Munster, Germany) attached to fluorescence microscope (Olympus, Hamburg, Germany) and cell nuclei as well as the number of γH2AX foci per cell were counted.

### 4.10. Statistics

Data are presented as means ± SE. Statistics were performed with GraphPad prism (GraphPad Software, San Diego, CA, USA). Significance threshold was set to *p* ≤ 0.05. Applied statistical tests are indicated in the figure legends.

## 5. Conclusions

The present study demonstrates radiogenic activation of K_Ca_3.1 channels in the MMTV-PyMT breast cancer model in vitro and in vivo for its contribution to oncogenic Ca^2+^ signals, DNA damage response and radioresistance. This radioresistance-conferring together with the reported tumor growth-promoting and the yet speculative metastasis-facilitating roles of the channel proposes K_Ca_3.1 as a new target during fractionated radiation therapy of breast cancer and the maintenance therapy thereafter. TRAM-34 and other K_Ca_3.1 inhibitors were proven efficient and selective in animal models. Among those, senicapoc was well tolerated with manageable side effects in clinical trials on sickle cell anemia [48,49]. Thus, K_Ca_3.1 targeting would be clinically feasible.

## Supplementary Materials

The following are available online at https://www.mdpi.com/2072-6694/11/9/1285/s1, Figure S1: Anti-cancer chemotherapeutics inhibit proliferation of MMTV-PyMT breast cancer cells independent from K_Ca_3.1.; Figure S2: Pharmacological blockade of K_Ca_3.1 channels does not improve the anti-cancer actions of chemotherapeutics in MMTV-PyMT breast cancer cells; Figure S3: Breast cancer MMTV-PyMT WT cells functionally express TRAM-34-sensitive, inwardly rectifying intermediate conductance and voltage-independent ion channels; Figure S4: [Ca^2+^]_i_ signals in MMTV-PyMT breast tumor cells depend on K_Ca_3.1 channels.; Figure S5: Radiation dose-response of MMTV-PyMT breast tumors and body weight.
