# Supplementary material for: KCa3.1 Channels Confer Radioresistance to Breast Cancer Cells
Source: Cancers (Basel). 2019 Sep 1;11(9):1285. doi: 10.3390/cancers11091285 (PMC6770875; doi:10.3390/cancers11091285)
Supplement: Supplementary file 1 [file cancers-11-01285-s001.pdf]

Supplementary Materials: KCa3.1 Channels Confer Radioresistance to Breast Cancer Cells

Corinna J. Mohr, Dominic Gross, Efe C. Sezgin, Friederike A. Steudel, Peter Ruth, Stephan M. Huber and Robert Lukowski

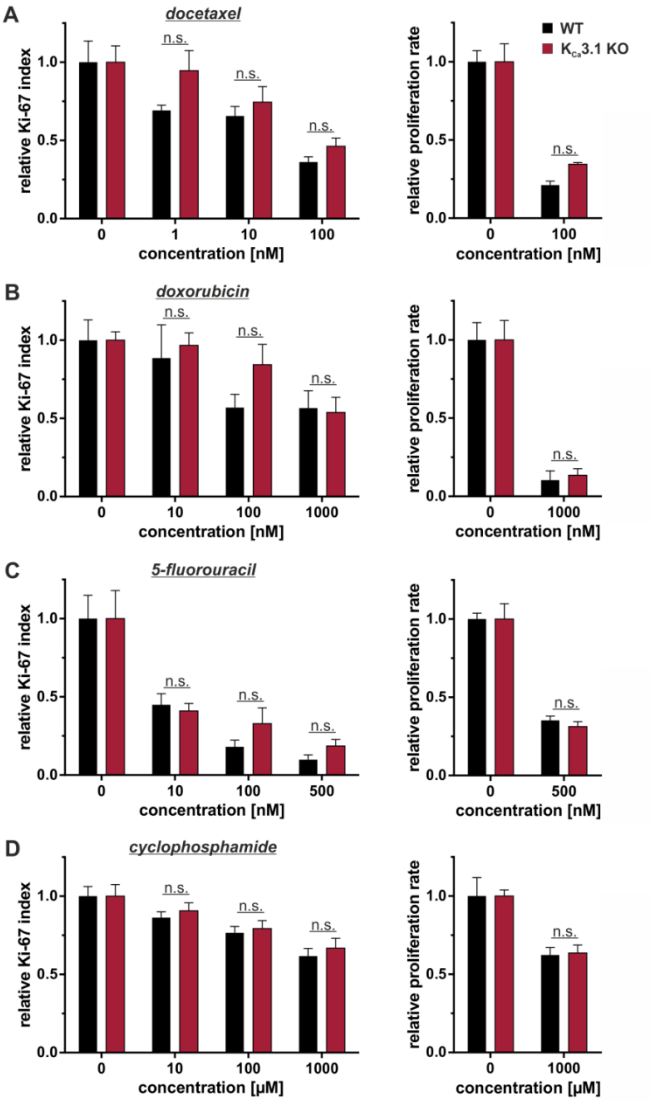

Figure S1. Anti-cancer chemotherapeutics inhibit proliferation of MMTV-PyMT breast cancer cells independent from KCa3.1. Effect of increasing concentrations of (**A**) docetaxel, (**B**) doxorubicin, (**C**) 5-fluorouracil or (**D**) cyclophosphamide on proliferation of MMTV-PyMT KCa3.1 WT (black bars) and KO (red bars) cells. Proliferation was assessed with immunofluorescence by Ki-67 index (**A–D,** left plots) and histologically by grid-based cell count (**A–D,** right plots). Data are shown as the means ± SE (n = 4–5) after normalization to the respective control conditions. *n.s.* indicates non-significant difference between treatment groups as determined by two-way ANOVA with Sidak's post-hoc test.

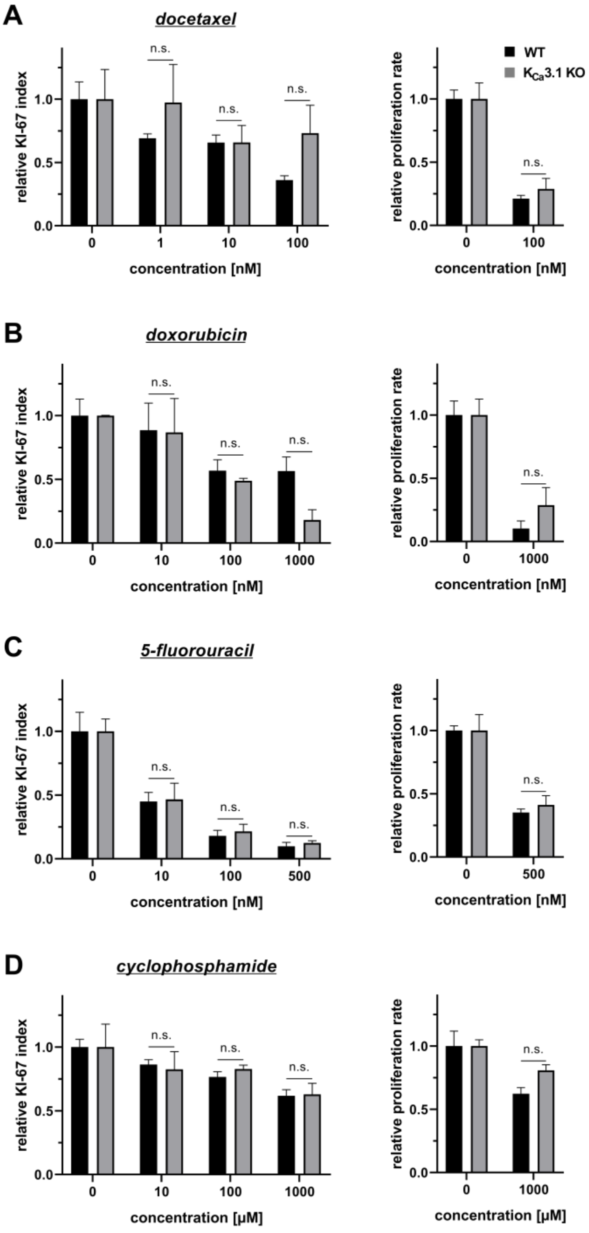

Figure S2. Pharmacological blockade of $K_{Ca}3.1$ channels does not improve the anti-cancer actions of chemotherapeutics in MMTV-PyMT breast cancer cells. Effect of increasing concentrations of (**A**) docetaxel, (**B**) doxorubicin, (**C**) 5-fluorouracil or (**D**) cyclophosphamide on proliferation of MMTV-PyMT $K_{Ca}3.1$ WT cells, which were either left untreated (black bars) or challenged with 10 µM TRAM-34 (grey bars). Proliferation was assessed with immunofluorescence by Ki-67 index (**A-D**, left plots) and histologically by grid-based cell counts (**A–D**, right plots). Data are shown as the means ± SE (n = 3–5) after normalization to the respective control conditions in the absence of TRAM-34 and chemotherapeutics. *n.s.* indicates non-significant difference between treatment groups as determined by two-way ANOVA with Sidak's post-hoc test.

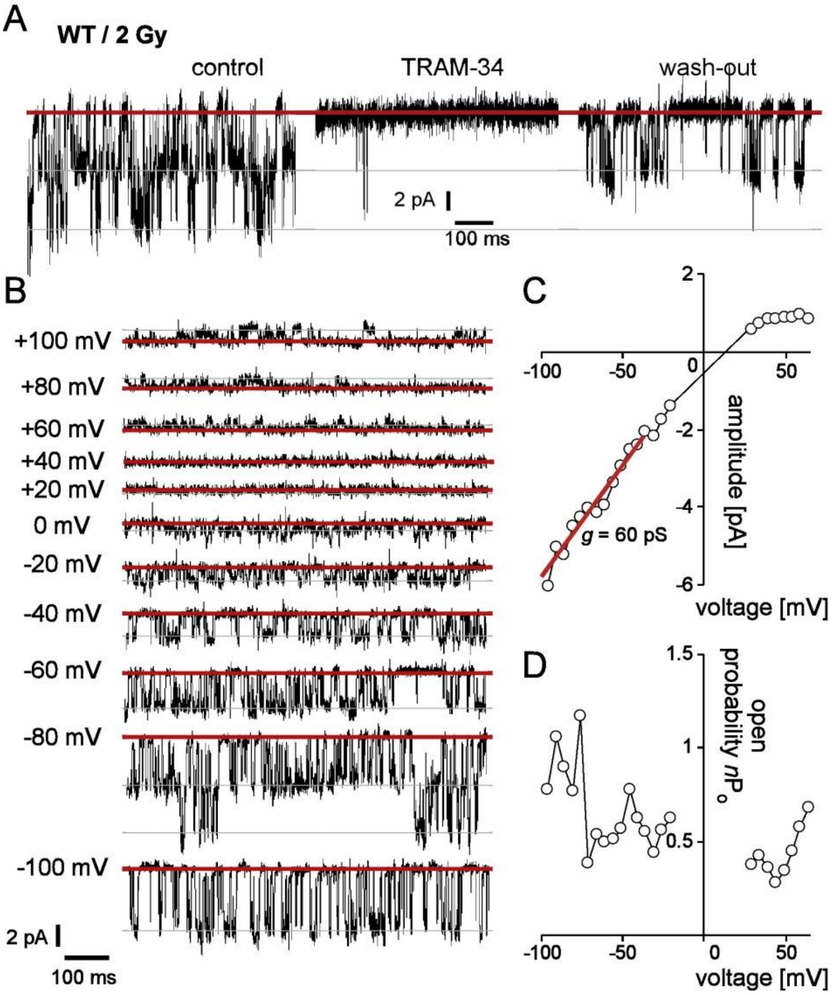

Figure S3. Breast cancer MMTV-PyMT WT cells functionally express TRAM-34-sensitive, inwardly rectifying, intermediate conductance and voltage-independent ion channels. (**A**) Single channel current transitions recorded in on-cell mode at −100 mV holding potential with KCl pipette- and NaCl bath solution from a 2 Gy irradiated MMTV-PyMT WT cell (320 min post irradiation) before, during, and after (wash-out) of TRAM-34 (2 µM). The red and grey lines indicate zero current and distinct unitary current levels. (**B**) Current tracings recorded at different holding potential as indicated. (**C**) Dependence of the unitary current transition (amplitude) and (**D**) open probability (nP_o) on holding potential.

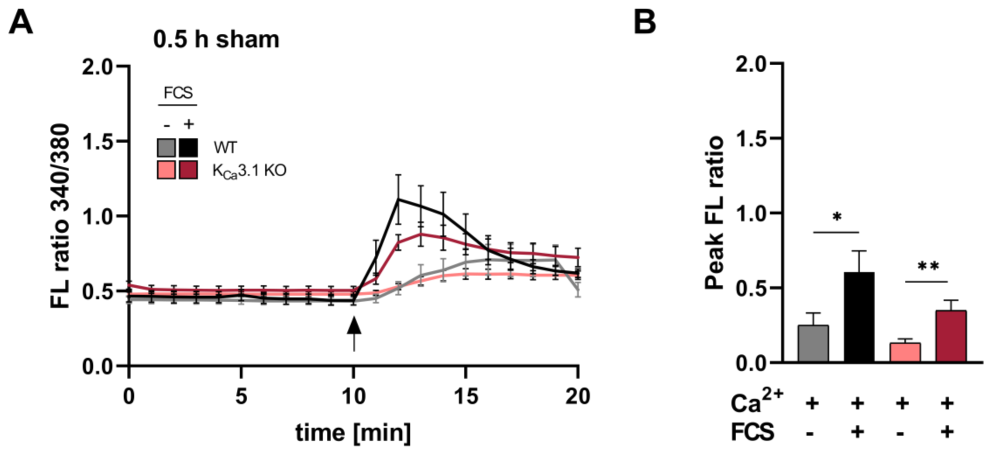

Figure S4. $[Ca^{2+}]_i$ signals in MMTV-PyMT breast tumor cells depend on $K_{Ca}3.1$ channels. (**A**) Acute Ca^{2+} signals of MMTV-PyMT mammary tumor cells evoked by Ca^{2+} (1.8 mM) only (grey, light red lines) or Ca^{2+} (1.8 mM) plus 5% FCS (black, red lines) after 30 min of sham treatment. FURA-2-AM-loaded cells were monitored in Ca^{2+}-free buffer for 10 min prior to the respective treatments as indicated. Arrow indicated buffer replacement. (**B**) The mean peak fluorescence (FL) ratio of the 340/380 nm wavelength was determined relatively to the respective baseline FL ratio for non-irradiated MMTV-PyMT $K_{Ca}3.1$ KO (n = 11–21 cells per condition derived from three different breast tumors) and WT (n = 9–15 cells per condition derived from three different breast tumors) groups. Statistical analysis was performed by two-way ANOVA with * and ** indicating $p \leq 0.05$ and $p \leq 0.01$, respectively, significant difference between genotypes.

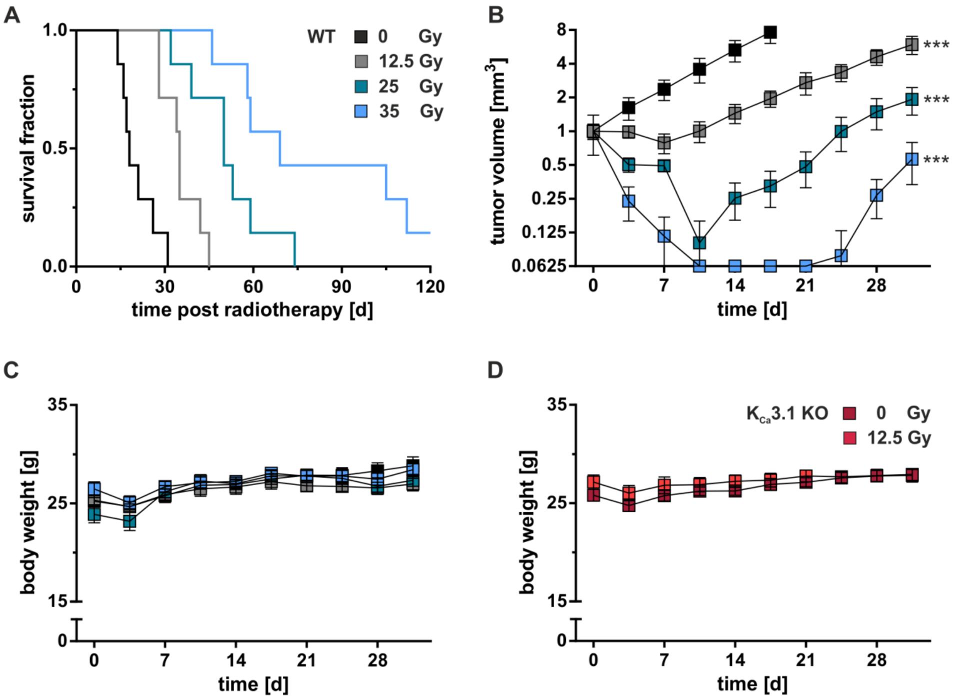

Figure S5. Radiation dose-response of MMTV-PyMT breast tumors and body weight. (**A**) Radiosensitivity of MMTV-PyMT KCa3.1 WT tumors in WT mice was tested by delivery of 12.5, 25 or 35 Gy in five fractions. Each individual dose regime led to a prolonged survival time as compared to unirradiated tumors (0 Gy). (**B**) Increase in tumor volumes was significantly delayed in the irradiated tumors as compared to the control situation (0 Gy). *** indicates $p \leq 0.001$ determined from two-way repeated measures ANOVAs and Tukey´s test. Due to its significant but intermediary effect, 12.5 Gy radiation dose was chosen for further experiments. MMTV-PyMT (**C**) WT or (**D**) KCa3.1 KO tumor-bearing WT mice did not differ in their body weight during the first four weeks after start of radiotherapy. Shown are means ± SE of $n = 7$ experiments in (**A**).
